# Frame‐based radiosurgery of multiple metastases using single‐isocenter volumetric modulated arc therapy technique

**DOI:** 10.1002/acm2.12672

**Published:** 2019-07-22

**Authors:** Kang‐Hyun Ahn, Kamil M. Yenice, Matthew Koshy, Konstantin V. Slavin, Bulent Aydogan

**Affiliations:** ^1^ Department of Radiation Oncology University of Illinois Chicago IL USA; ^2^ Department of Radiation and Cellular Oncology University of Chicago Chicago IL USA; ^3^ Department of Neurosurgery University of Illinois Chicago IL USA

**Keywords:** frame‐based SRS, multiple metastases, single‐isocenter volumetric modulated arc therapy, VMAT SRS

## Abstract

Single‐isocenter volumetric modulated arc therapy (VMAT) technique can provide stereotactic radiosurgery (SRS) treatment with improved delivery efficiency for treating multiple metastases. Nevertheless, planning is time consuming and verification of frame‐based SRS setup, especially at noncoplanar angles, can be challenging. We report on a single‐isocenter VMAT technique with a special focus on improving treatment workflow and delivery verification to exploit the minimized patient motion of the frame‐based SRS. We developed protocols for preplanning and verification for VMAT and evaluated them for ten patient cases. Preplans based on MRI were used to generate comparable treatment plans using CT taken on the day of treatment after frame placement. Target positioning accuracy was evaluated by stereoscopic in‐room kV imaging. Dosimetric accuracy of the noncoplanar plan delivery was validated using measurement‐guided 3D dose reconstruction as well as film‐based end‐to‐end test with a Rando phantom. Average absolute differences of homogeneity indices, conformity indices, and V12Gy between MR preplans and CT‐based plans were within 5%. In‐room imaging positioning accuracy of 0.4 mm was verified to be independent of the distance to the isocenter. For treatment verification, average local and global passing rates of the 3D gamma (1 mm, 3%) were 86% and 99%, respectively. D99 values were matched within 5% for individual target structures (>0.5 cc). Additional film analysis confirmed dosimetric accuracy for small targets that had large verification errors in the 3D dose reconstruction. Our results suggest that the advantages of frame‐based SRS and noncoplanar single‐isocenter VMAT technique can be combined for efficient and accurate treatment of patients with multiple metastases.

## 1 INTRODUCTION

Brain metastases are the most common intracranial neoplasms that require precise treatment modalities to achieve high local control. Previous clinical studies validated the effectiveness of stereotactic radiosurgery (SRS) in the treatment of brain metastases and showed that various commercially mature solutions could deliver cranial SRS with a high level of geometrical and dosimetric accuracy.^1^, ^2^, ^3^ These developments resulted in a paradigm shift toward definitively treating an ever increasing number of targets with radiosurgery. Treatment of multiple metastases, with traditional linac‐based SRS approach still presents challenges due to significant effort in planning and delivering treatments to multiple isocenters.

Recent studies demonstrated that a single‐isocenter technique combined with volumetric modulated arc therapy (VMAT) optimization for planning could be used to generate clinically acceptable plans that were comparable to the conventional multi‐isocenter approach.^4^, ^5^, ^6^, ^7^, ^8^ The single‐isocenter VMAT technique substantially lowers the beam‐on time without compromising plan qualities such as dose fall‐off and conformity.^6^, ^9^, ^10^ In particular, the VMAT solution by Varian known as RapidArc was used by several investigators to achieve highly conformal dose distributions with optimal treatment delivery efficiency for multiple lesions. Since VMAT planning is rather complex with multiple targets and organs at risk for optimization, it typically uses a workflow that employs a mask immobilization and CT simulation followed by several days of planning before the patient is treated. The frameless technique typically involves 1–3 mm planning target volume (PTV) margin.^11^, ^12^, ^13^ In particular, rotational errors for targets at distances away from the isocenter would negatively affect the treatment delivery accuracy unless an additional margin is added due to the less effective immobilization and setup with a nonrigid mask system.^14^, ^15^, ^16^ Also, accurately positioning the patient for treatment setup for all treatment couch angles becomes the challenge for this approach since multiple targets are treated with one isocenter. A more rigid patient immobilization and setup afforded by traditional invasive stereotactic frame would minimize any potential patient motion and eliminate rotational misalignments during treatment increasing the effectiveness of single isocenter VMAT SRS for multiple targets. This requires the complex VMAT planning and QA process to be carried out in a same day procedure of Computed Tomography (CT) simulation and treatment with less than 8 h of time window. Therefore, a preplanning is crucial to frame‐based VMAT SRS to address planning challenges before the treatment day.

We devised a practical approach to improve workflow and also established the delivery accuracy of single isocenter VMAT for frame‐based SRS of multiple metastases. We utilized Magnetic Resonance Imaging (MRI) scans of SRS patients, which are acquired 2–3 days before the frame placement for target definition, preplanning, and treatment optimization. We also validated the spatial accuracy of in‐room kV imaging system combined with 6 degrees of freedom couch to be insensitive to the distance between the isocenter and each of multiple targets, and minimized the delivery time of noncoplanar beams using the image‐based target positioning. The performance of patient‐specific treatment verification was evaluated using patient geometry dose reconstructed from diode detector array measurements. This allowed dosimetric analysis for individual targets as well as 3D gamma evaluation for the acceptability of the entire plan.

## 2 METHODS

RapidArc plans were prepared using Varian External Beam Planning version 13.7 and delivered on a TrueBeam radiotherapy system equipped with a Millenium 120 MLC (Varian, Palo Alto, CA, USA). We evaluated patient‐specific preplanning for ten patient cases with 3–9 intracranial lesions, 57 targets in total, which were treated at our institution over the past 2 yr as described below. Preplans were generated using the body contours and target volumes as defined in axial MR images. For the calculation of dose, Hounsfield unit of zero was assigned to the MR body contour. Target volumes ranged 0.03–20 cc with the prescription doses of 14–18 Gy. All plans had 360‐degree axial arcs and 180‐degree vertex arcs at two couch angles. Each arc was paired with one having an opposite gantry rotation and an orthogonal collimator angle. The plans were generated with the 6‐MV flattening filter free beam at a dose rate of 1400 monitor units (MU) per minute using Photon Optimizer, Smart LMC, and Anisotropic Analytical Algorithm with 1.0‐mm grid. Normal tissue objective was turned on and concentric optimization structures were created surrounding each target to maximize dose fall‐off and minimize normal brain dose at V12Gy level.^6^ Optimization objectives were adjusted to cover 99% of the gross target volume with the prescription dose while limiting the dose to any critical organs near the target volumes. Tumor volumes were primarily defined on the MR imaging which was always acquired within less than a week of the SRS delivery. On the SRS procedure day, MR information was registered with the spatially accurate CT which is localized in the stereotactic reference system to ensure accurate target localization. Then, the plan was re‐optimized using the beam settings and constraints set retrieved from the preplan. With the same target volume coverage as that of the preplans, homogeneity indices (HI, maximum dose/prescription), conformity indices (CI, ICRU 62 definition),^18^ and V12Gy were compared to evaluate the reproducibility of the plan quality. The MR images had slice thickness and in‐plane resolution values ranging from 0.4 to 1.4 mm, while the SRS planning CT scans had isotropic resolution of 0.7 mm.

The treatment delivery used the PerfectPitch 6 degrees of freedom couch on TrueBeam combined with in‐room kV x‐ray imaging of ExacTrac system (Brainlab, Munich, Germany) for target positioning. ExacTrac x‐ray positioning tolerance was set to the minimum (0.2 mm), and the “reference star” array was used to relay the couch correction information. The robotic positioning performance of the system was evaluated using orthogonal MV images of a polystyrene phantom with five imbedded radiopaque markers at arbitrary distances from the isocenter. The phantom had metallic wires wound in an arbitrary shape to drive the image guidance. The Digital Megavolt Imager of TrueBeam acquires 1280 × 1280 pixels over 43 cm × 43 cm field size, which defines 0.22‐mm resolution with the source‐to‐detector distance of 150 cm. The positioning error was defined as the distance discrepancies of the centers of BB's between the digitally reconstructed radiography (DRR) and the direct imaging with the therapeutic MV beams.^19^ The distances were measured using the Varian Offline Review tools.

Dosimetric accuracy of the RapidArc plans was verified using a measurement‐guided 3D patient dose with 1‐mm grid reconstructed by ArcCHECK 3D diode detector array (Sun Nuclear, Melbourne, FL, USA) and 3DVH version 3.2. Briefly, the entrance and exit absolute dose measurements are summed into a 3D composite dose within the cylindrical volume of the ArcCHECK, which is subsequently morphed onto the patient geometry.^20^ The measurement required noncoplanar couch angles to be reset to 0°, but the software reconstructed the 3D dose distribution of the original noncoplanar configuration from the coplanar measurement. Error specificity of the verification was evaluated using selected treatment plans cast on a Rando head phantom. Dosimetric errors between calculated plans and delivery measurements were induced by applying −6% to +6% normalizations to the intended plans. For example, dose measurement of an 18‐Gy plan underwent verification analysis using the original plan (0% error) as well as plans normalized from the original treatment plan to deliver 16.9, 17.5, 18.5, and 19.1 Gy to the target volume, which correspond to −6%, −3%, 3%, and 6% induced errors, respectively. Also, to further evaluate the target size dependency of the error specificity, we prepared treatment plans treating four structure sets of Rando phantom: CT0.1cc, CT0.5cc, CT1cc, and CT5cc. Each structure set had four spherical targets of the same size as denoted in the label.

We carried out the coplanar verification measurements for the ten patients, and evaluated 3D gamma passing rates at 1‐mm distance and 3% dose difference with a 15% threshold for each patient plan as well as the D99 (dose that covers 99% of the PTV) for each target. We also randomly selected one patient plan and performed further film analysis to further study the dose statistics reported by 3DVH calculations. The corresponding plan was transferred to the Rando phantom with an adjustment of isocenter so that the target of evaluation falls on embedded Gafchromic EBT3 films. In order to do the film analysis, we normalized the prescription dose so that delivered dose could be appropriately assessed in the calibrated range of the film (<10 Gy). We followed the same imaging and positioning protocol for the film dosimetry as for patients, that is, delivering the noncoplanar “treatment” plan. Film dosimetry was performed using a home‐built python script following the suggestions of previous studies.^21^, ^22^ Briefly, an EPSON Expression 10000XL scanner was used in 48‐bit RGB mode at a resolution of 150 dpi, 24‐h after irradiation. We used 8 cm × 8 cm films with a consistent orientation, and placed them at a reproducible central location of the scanner to minimize the lateral dependence artifacts. We calibrated the optical density range 0–10 Gy using a weighted sum of the three channels of red, green and blue. Institutional Review Board approval was obtained for this study.

## 3 RESULTS

**Figure 1:**
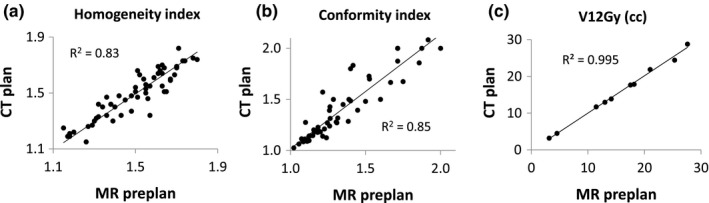
Scatter plots of dosimetric indices. Differences between MR and CT plans are inevitable due to discrepancies of Hounsfield unit and axial plane orientation. Nevertheless, MRI preplan performances were comparable to CT plan as evaluated with (a) homogeneity, (b) conformity, and (c) normal brain volume receiving 12 Gy

**Figure 2:**
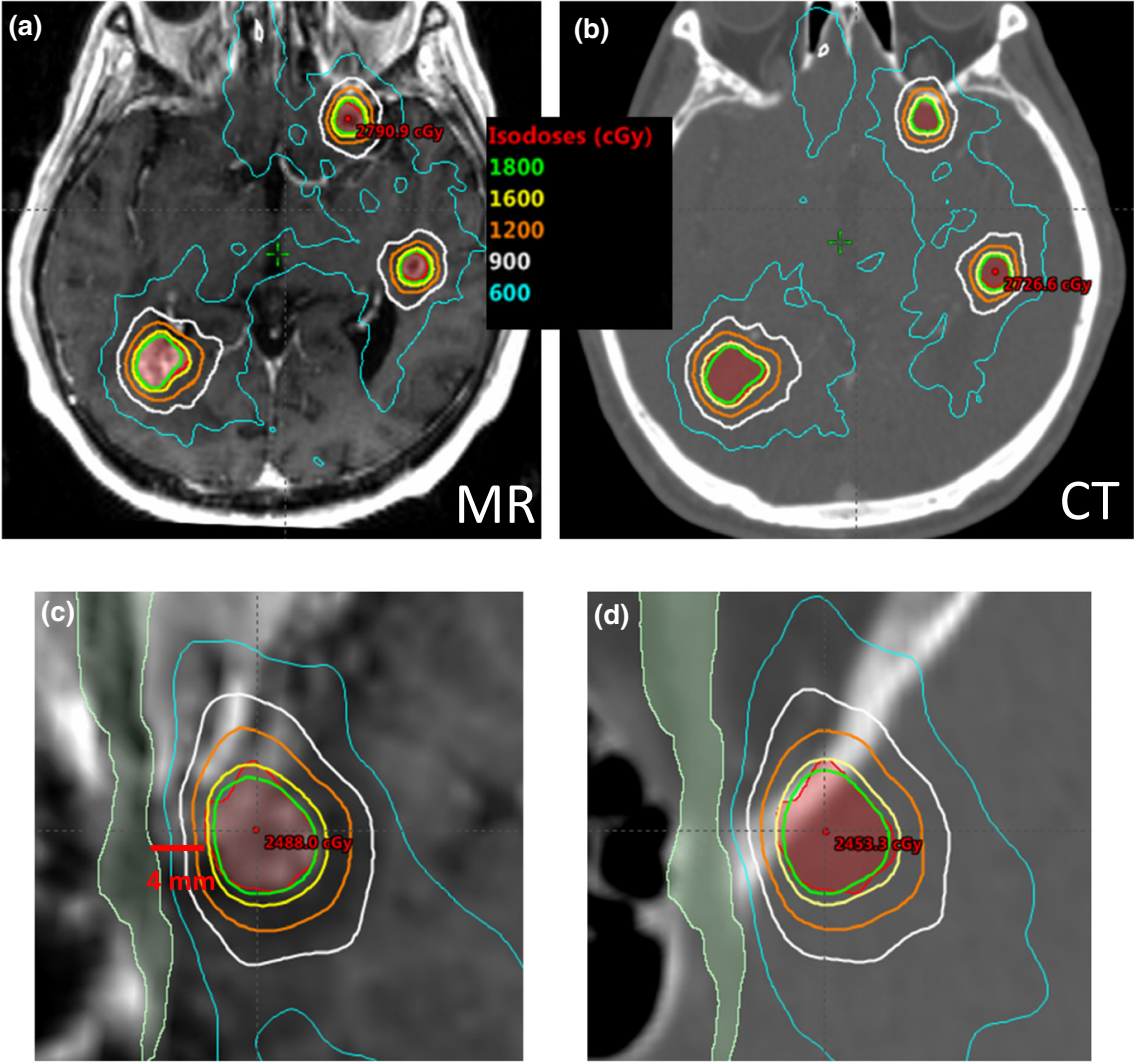
Dose distribution comparison of MR preplan and CT plan. The shape of isodose lines of the MR preplan in (a) and (c) were closely matched to those of the CT plan in (b) and (d). Preplanning enables thorough exploration of optimization performance, and can be of particular help to handle patient‐specific challenges ahead of the frame placement. Bottom images ((c) and (d)) show the close‐up dose distributions for a target volume (red) near the optic nerve (light green)

The clinical plan qualities for all MR‐based preplans and stereotactic CT plans were comparable in terms of target coverage statistics and normal tissue dose. Table 1 lists median and range of the dosimetric indices for the 57 targets in ten patients in this study. Homogeneity and conformity were evaluated for each target, and normal brain volume receiving 12 Gy (V12Gy) was calculated for each patient. Figure 1 shows the scatter plots of homogeneity index (HI), conformity index (CI), and noninvolved brain V12Gy. Overall, the target HI and CI as well as the normal brain V12Gy varied depending on the size, shape, and number of target volumes, but the CT plans reproduced similar values as those calculated with MR preplans with average absolute differences of 5.2%, 3.6%, and 2.1%, respectively. Figure 2 shows an example of dose distributions at an axial plane that intersects three target volumes (red). The shape of isodose lines of the MR preplan were closely matched to those of the CT plan. In particular, as one of the target volumes was near the optic nerve, the constraint set, and the prescription dose were customized to protect the critical organ. The two bottom panels show close‐up views of the planar dose distributions for the optic nerve and the nearby target.

**Table 1:** Median and range of dosimetric indices for 57 targets in 10 patients

	Homogeneity index	Conformity index	V12Gy
MR preplan	1.52 [1.15–1.80]	1.25 [1.02–2.00]	15.83 [3.15–27.6]
CT plan	1.51 [1.15–1.82]	1.27 [1.03–2.08]	15.77 [3.19–28.8]

**Figure 3:**
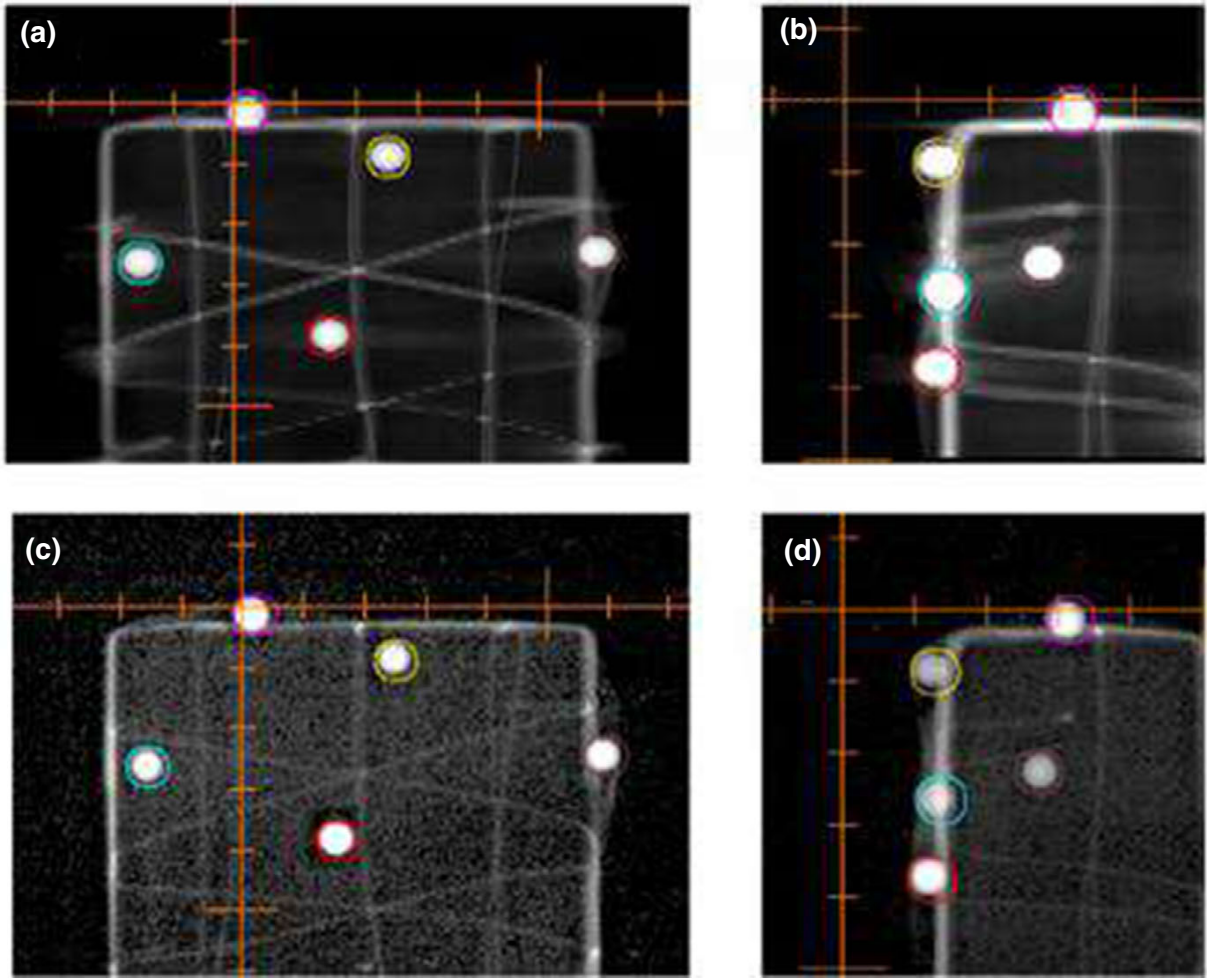
Demonstration of ExacTrac/PerfectPitch positioning accuracy with AP (left; (a) and (c)) and lateral (right; (b) and (d)) views of the multiple target BB phantom. Circular contours were generated from the CT. Positioning accuracy was defined as distance discrepancies between the 5‐mm diameter BB's of DRR (top; (a) and (b)) and of images from the therapeutic MV beams (bottom; (c) and (d))

**Figure 4:**
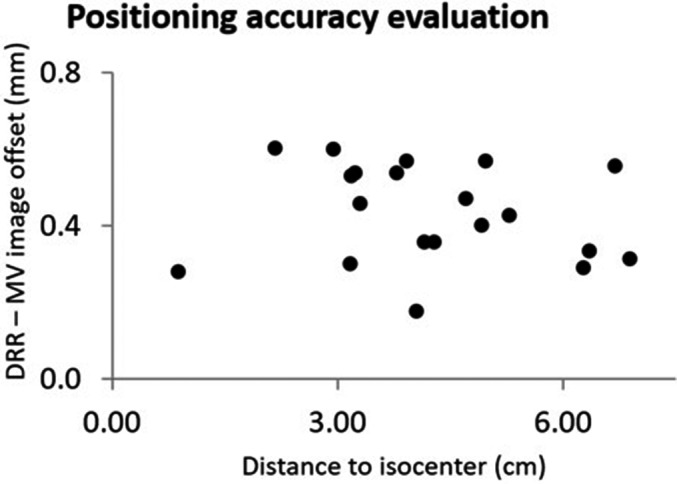
ExacTrac/PerfectPitch positioning accuracy. Average offsets between the planned and imaged BB positions were 0.4 mm, and had negligible correlation with the distance to isocenter (r^2^ < 0.1)

Figure 3 shows AP (left; a and c) and lateral (right; b and d) views of the multiple target BB phantom. Top panels (a and b) are DRR's, and the bottom panels (c and d) are MV images acquired after 6D ExacTrac correction was applied with the PerfectPitch couch. Distance offset of the BB centers between the DRR's and the MV images demonstrated the performance of the positioning system, which was estimated to be 0.4 mm. The distance measurements were repeated for 20 arbitrary BB positions, and are summarized in Fig. 4. The average and standard deviation of the distance discrepancies were 0.44 and 0.13 mm. Correlation with the distance to the isocenter was negligible (r^2^ < 0.1).

**Figure 5:**
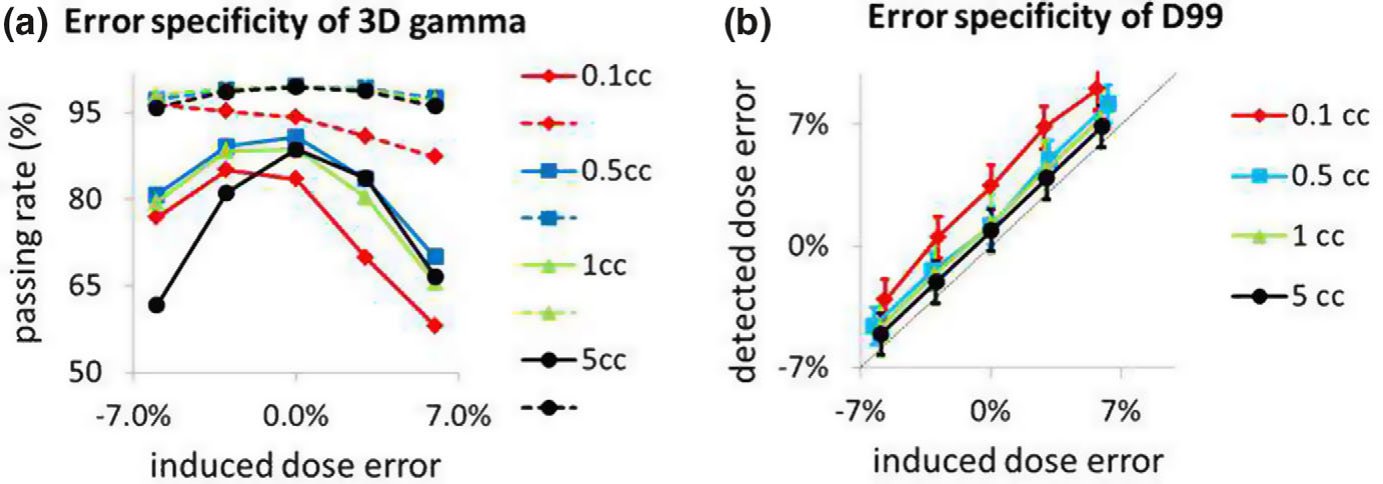
Responses of 3DVH dose reconstruction to induced errors. Each plan treated a structure set having four spherical targets of the same size. Four such structure sets were created to evaluate the effect of target sizes (0.1–5 cc). (a) 3D gamma passing rates. Global gamma (dotted line) had poor specificity to induced errors. Although local gamma (solid line) was more responsive to the induced errors, the passing rates were subject to target‐size dependency. (b) D99 had linear responses to the induced dose errors, and indicates that they could supplement gamma analysis

Figure 5 shows the specificity of 3DVH software to the artificially created dosimetric errors in the Rando phantom. Positive values of the induced error correspond to delivery of higher dose than planned. Overall, the 3D gamma passing rates [Fig. 5(a)] decreased with the induced errors. Global gamma (dotted lines), however, did not change much over the error range, and reported 95% passing rates even to a 6% dosimetric delivery error. Local gamma evaluations (solid lines) had increased failing points mostly around low dose area outside the targets, but they were more responsive to the introduced delivery errors. There was another complication, and the passing rates had a variation dependent on target sizes. Note that the plan treating tiny targets (0.1 cc) without any induced error reported local gamma passing rates (~80%) comparable to the plan treating 0.5–1 cc targets with −6% delivery error. D99 values from individual structures in Fig. 5(b) had variations linearly correlated with the induced dose errors. There was an unresolvable bias, which was aggravated for the plan treating 0.1‐cc targets.

**Figure 6:**
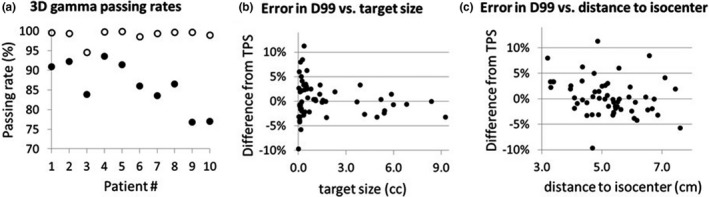
ArcCHECK/3DVH verification of 10 patients. (a) Global (open circle) and local (solid circle) 3D gamma passing rates. (b) D99 discrepancies of 3DVH and TPS for individual targets were < 5% for target sizes> 0.5 cc. (c) D99 discrepancies were not correlated with the distance from the isocenter (r^2^ < 0.1)

Figure 6 shows verification results of the ten patients. Average local and global passing rates of the 3D gamma [Fig. 6(a)] were 86.2% and 99.0% with the standard deviations of 6.0% and 1.6%, respectively. Two patients had local gamma passing rates lower than 80%. There were 15 targets in them with ten targets less than 0.5 cc, and four small targets had D99 discrepancies greater than 5%. Figures 6(b) and 6(c) show errors in D99 for the ten patients plotted against target size and the distance to isocenter, respectively. Overall, large errors reported by 3DVH verification were associated with small targets. For targets greater than 0.5 cc, D99 values agreed with the treatment plan within 5%. No overall trends were found with the distance to isocenter (r^2^ < 0.1).

**Figure 7:**
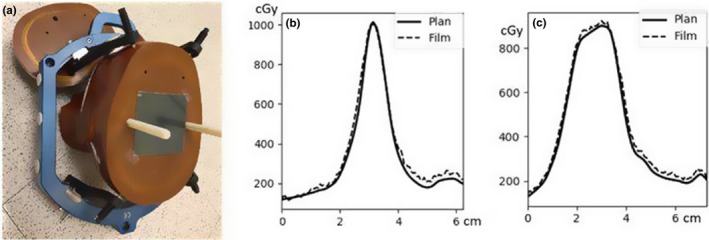
Two targets (0.4 cc, 5.2 cc) were selected for end‐to‐end test. Isocenter was adjusted so that the target of evaluation falls on the embedded Gafchromic EBT3 film as shown in (a). Although 3DVH reported 11.2% and 0.3% discrepancies from TPS for the 0.4‐cc and 5.2‐cc targets, respectively, film measurements were within 2% of TPS for both targets. (b) Plan vs film for 0.4‐cc target. (c) Plan vs film for 5.2‐cc target

The tenth patient in the left panel of Fig. 6 was selected for further investigation. This patient had eight targets ranging 0.1–8.4 cc, and five targets were less than 0.5 cc. We performed film‐based end‐to‐end test for two targets. Their volumes were 0.4 and 5.2 cc, and the values of D99 reported by 3DVH were 11.2% and 0.3% higher than that of treatment planning system (TPS), respectively. Figure 7 shows the film setup and measurements of each target compared to the TPS and 3DVH. Contrary to the 3DVH dose reconstruction results, both targets had measured dose values within 2% of TPS.

Overall, treatment planning and verification were usually finished within 2–3 h on the day of SRS procedure for most patients. Target positioning took less than 30 min, and the treatment typically had beam‐on time <10 min at 1400 MU/min.

## 4 DISCUSSION

In treating patients with multiple metastases, applying the single isocenter technique to frame‐based SRS presents unique challenges to overcome. With the placement of the invasive head ring, the SRS workflow is subject to rigid time constraints, and the computation‐intensive VMAT planning could substantially impede the procedures. Patient MRI scans are usually acquired a few days before the treatment, and enable treatment planning rehearsal in advance. However, in reality, the axial plane orientation of VMAT is determined after the frame placement on the SRS day, and it can only be approximated with the MRI scans. As shown in Fig. 1, it turns out that the planning performance is rather insensitive to such discrepancies of orientation with the combined use of axial and vertex arcs, and orthogonal pairs of collimator angles within each arc.

Often, the patient‐specific plan performance limits are gradually revealed to planners with iterative processes of plan optimization and evaluations. Furthermore, depending on the anatomical difficulties involved, full exploitation of the trade‐off between multiple target coverages and critical structure sparing might require an indefinite amount of planning time especially with the unknown performance limits. Our results indicate that treatment goals, or performance limits, set at MRI preplanning phase could be closely reproduced in the plans on the CT scans. Consequently, the time‐consuming processes of exploring attainable treatment goals can practically be channeled into preplanning stage, thus substantially facilitate the workflow on the treatment day.

Figure 2 shows an example where the MRI preplanning can further benefit the treatment. One of the multiple targets happened to be 4‐mm from the optic nerve, and the dosimetric gradient between the target and the critical organ was thoroughly evaluated with the MRI preplanning to work out an acceptable level of prescription dose. In applying VMAT technique to frame‐based SRS, it is critical to communicate patient‐specific challenges with physicians in advance. If any adjustment of treatment strategy is required, this needs to be discussed before the placement of the invasive head ring on the patient.

The short beam‐on time (<10 min) of single‐isocenter VMAT SRS might indicate reduced intra‐fractional error for a frameless setup.^12^ Nevertheless, the total treatment time including patient positioning for noncoplanar setup could still allow small motions under the mask, which would be amplified further by rotational errors especially for targets away from the isocenter. While single‐isocenter VMAT facilitates the dose delivery in a short time, frame‐based SRS provides additional safety for integrity of treatment setup by eliminating intra‐fractional motion, and could maximize the therapeutic ratio with optimal and safe treatment. At the same time, it can severely penalize the treatment outcome even with a slight error in positioning. Therefore, it is critical to verify the spatial positioning accuracy with a minimum possible tolerance for a frame‐based technique, and also to assess any variation with the distance to the isocenter if multiple targets are handled by a single isocenter. Figure 4 demonstrates that the positioning performance of the stereoscopic in‐room kV imaging was independent of the distance between the target and the isocenter. Furthermore, the average discrepancy of 0.4 mm between the DRR and the MV imaging indicates the highest attainable spatial accuracy with our setup, which is presumably limited by the intrinsic accuracy of DRR generated by the CT with the minimum slice thickness of 0.7 mm.

Frame‐based immobilization is a mature technique and no additional treatment margin is needed for a target treated at the isocenter.^24^, ^25^ We used a dedicated target‐positioning box provided by the manufacturer of our SRS positioning system for the initial patient setup and quality assurance of our single‐isocenter multiple target treatments. We verified the patient setup further with image‐ guidance and confirmed that the spatial accuracy was consistently maintained at distances away from the isocenter for all targets with our frame‐based setup. We point out, however, if a clinic attempts to use frame only setup without any image guidance, a careful assessment of rotational error should be carried out to determine corresponding treatment margins due to any possible residual rotations away from the isocenter. Any rotational error, however, intrinsically penalizes the smaller initial PTV margin by requiring a relatively larger additional margin for the off‐isocenter targets.^17^ Addition of extra margins would defeat the purpose of the invasive stereotactic frame placement, and may lead to a detrimental reduction in therapeutic ratio by increasing the risk of necrosis of normal brain tissue.^23^

Volumetric modulated arc therapy involves delivery of inversely optimized dynamic MLC while gantry is in motion, which requires a patient‐specific verification measurement. There are several conditions pertinent to this treatment modality. First, the verification needs to be performed within the limited time window of the frame‐based workflow. For SRS, it is desirable to use measurement devices that would support a gamma analysis with at least 1‐mm tolerance level. On the other hand, as discussed below, gamma passing rates may be suboptimal to assess a plan treating multiple metastases. Using ArcCHECK and 3DVH, we performed 3D gamma analysis in the patient geometry as well as dosimetric evaluation for individual target volumes.

The responses of 3DVH to the induced errors as shown in the phantom study (Fig. 5) demonstrates the complexity associated with the plan verification. Global gamma had poor specificity to the induced delivery errors, and was not acceptable for our purpose. Local gamma analyses were compounded by target size dependency. Using passing rates alone, it would be difficult to identify a potential delivery error with the realistic cases involving different target sizes. The linear response of D99 values to the induced errors indicates that the verification could be augmented by investigating reconstructed dosimetric parameters of individual targets.

According to our phantom study, patients with local gamma passing rates >85% would correspond to delivery verification within 5% regardless of target volume size. The dosimetric quality range of ±5% is considered as clinically insignificant incidents.^26^, ^27^ Lower passing rates would require further investigation of individual target coverage. However, the reconstructed D99 values collected over the ten patients had substantial discrepancies from TPS with up to 10% scatters. The magnitudes of these errors were independent of the distance to isocenter (Fig. 6c), which is consistent with the positioning accuracy analysis in Fig. 4. Instead, they were strongly correlated to the target sizes as shown in Fig. 6(b). The independent film measurements (Fig. 7) confirmed that these errors were not real, and they may represent the inherent limitation of the ArcCHECK/3DVH performance for small targets.

Accurate verification of small targets (<0.5 cc) within the limited time of treatment workflow may not be easy for most clinical setup. While high‐resolution film dosimetry is ideal for small target SRS verification, it does not lend itself for an efficient process for the same day SRS planning and delivery procedure. However, our film dosimetry analysis consistently showed that small target dose verification was within acceptable limits giving us the confidence in our VMAT SRS commissioning to use the lower‐resolution ArcCHECK measurements in the clinical setting for a more efficient QA process. On the other hand, addressing the uncertainties (~10%) of the target coverage with corresponding dose elevation is relatively more affordable considering the small volumes and the high dose gradient (~3 Gy/mm) of VMAT. In practice, 2‐Gy dose elevation at the target boundary would inflict sub‐millimeter expansion of the prescription isodose line. For example, as the tenth patient [Fig. 6(a)] had a questionable verification result, we re‐optimized it with 10% higher doses to five smaller targets (<0.5 cc). This increased V12Gy from 22.2 to 22.5 cc.

## 5 CONCLUSION

We devised a practical approach that can combine advantages of frame‐based SRS with noncoplanar single‐isocenter VMAT in treating multiple metastases. MRI preplanning using VMAT SRS technique and plan QA performed with ArcCHECK/3DVH allowed for efficient planning and accurate delivery verification within the limited time window. ExacTrac in‐room kV imaging expedited noncoplanar patient setup with the invariant spatial accuracy across multiple targets.

## CONFLICT OF INTEREST

No conflict of Interest.
